# A Fish Farmer's Encounter With Leptospirosis: A Case Report

**DOI:** 10.7759/cureus.48138

**Published:** 2023-11-02

**Authors:** Hamza Rashid, Ayooluwa K Omoloye, Siraj Y Abualnaja, Samson O Oyibo, Olugbenro O Akintade

**Affiliations:** 1 Medicine, Peterborough City Hospital, Peterborough, GBR; 2 General Medicine, Peterborough City Hospital, Peterborough, GBR; 3 Diabetes and Endocrinology, Peterborough City Hospital, Peterborough, GBR; 4 Elderly Care Medicine, Peterborough City Hospital, Peterborough, GBR

**Keywords:** anicteric hepatitis, icterohemorrhagic leptospirosis, weil's disease, occupational disease, fish-farmer, zoonotic infections, leptospirosis

## Abstract

Leptospirosis is a zoonotic infection primarily caused by bacteria of the genus *Leptospira. *This infectious disease mainly occurs through direct contact with infected animals or indirect contact via contaminated soil or water. While the incidence rate of leptospirosis in the developing world is as high as 100 cases per 100,000 population, the incidence rate in the United Kingdom is low (0.14 cases per 100,000 population). We present a 56-year-old male fish farmer who presented to the emergency department with a history of intense thigh pain and sudden inability to mobilise following a week-long period of a flu-like illness, characterised by worsening myalgia localised to the inner thighs, fever, and episodes of passing dark red urine. Initial investigations demonstrated acute renal impairment, hepatitis, thrombocytopenia, mild rhabdomyolysis and raised inflammatory markers. With a suspected diagnosis of leptospirosis after a detailed clinical history and preliminary blood tests, treatment was immediately commenced with intravenous antibiotics, intravenous rehydration and vigilant monitoring of urinary output. The patient’s condition rapidly improved and the diagnosis was later confirmed by a positive Leptospira polymerase chain reaction (PCR) report and serology. We believe prompt treatment prevented deterioration in this case. The aim of this case report is to highlight the importance of a detailed clinical history, with a particular focus on occupational exposure, especially in the developed world. Additionally, a low clinical threshold for leptospirosis is imperative, as rapid clinical deterioration can happen if no immediate medical intervention is performed.

## Introduction

Leptospirosis, primarily caused by spirochete bacteria of the genus *Leptospira*, often remains an underappreciated zoonotic concern. This infectious disease encompasses over 250 pathogenic serovars within the Leptospira genus, with transmission to humans occurring through direct contact with infected animals, particularly rodents and livestock, or indirectly via contaminated soil or water [1].

The global distribution of leptospirosis cases presents a stark contrast; for instance, mainland Africa reports an annual incidence of 75 to 102 cases per 100,000 population, whereas the United Kingdom reports a significantly lower rate of 0.14 cases per 100,000 population [2,3]. This divergence highlights the significant impact of socioeconomic and environmental variables, including occupation, access to sanitation, and regional rainfall patterns, in shaping the epidemiology of this disease.

Occupational factors emerge as prominent determinants of leptospirosis risk, notably affecting individuals engaged in sewage management, farmers with exposure to water bodies contaminated by the urine of rodents and livestock, as well as butchers handling the kidneys of infected animals [4].

The pathogenesis of leptospirosis infection begins with the spirochetes (leptospires) breaching the oral mucosa and entering the lymphatics and bloodstream. The incubation period is generally 7-14 days. Elevated levels of leptospiremia can trigger an immune response akin to sepsis, characterised by heightened levels of interleukin-6 (IL-6) and tumour necrosis factor-alpha (TNF-alpha) [5]. In its most severe form, leptospirosis can present as icterohemorrhagic leptospirosis (Weil's disease) accompanied by acute kidney injury, acute liver failure, rhabdomyolysis, thrombocytopenia, and respiratory failure [6]. These severe cases occur in approximately 5-10 per cent of symptomatic instances and are associated with a mortality rate ranging from 5 to 15 per cent [7].

From a diagnostic perspective, the initial screening test is the enzyme-linked immunosorbent assay (ELISA) to detect leptospira-specific antibodies. Confirmatory testing includes the microscopic agglutination test (MAT), which serves as the gold standard. Symptomatic patients typically receive a positive diagnosis when a more than fourfold increase in serum titres is observed over a 3-5-day interval. An alternative confirmatory test involves the use of polymerase chain reaction (PCR) assays; however, these assays lack the precision to pinpoint the specific infecting serovar. The transience of leptospires in body fluids means that a negative PCR test does not exclude leptospirosis [8]. Additionally, leptospires can be isolated and identified through blood culture media [5]. However, culture is insensitive and slow and is therefore not recommended as the sole diagnostic method.

Diagnosing leptospirosis presents challenges, as it shares non-specific symptoms with various other conditions, including influenza, malaria, yellow fever, and dengue. Therefore, it is imperative to establish clinical suspicion for leptospirosis early on, within the relevant epidemiological context. The importance of a comprehensive patient history, which includes occupational exposure cannot be overemphasized [4].

The literature provides ample evidence supporting early therapeutic intervention in severe cases, which can help shorten the duration of fever and reduce the risk of lasting organ damage [5]. The preferred therapy typically involves intravenous penicillin G, doxycycline or third-generation cephalosporins, such as ceftriaxone, a treatment option that has demonstrated comparable, if not superior, efficacy to intravenous penicillin [9].

## Case presentation

Medical history and demographics

A 56-year-old, male fish farmer presented to the emergency department with a history of intense thigh pain and sudden inability to mobilise. In the week proceeding this presentation, he had a flu-like illness, characterized by fever, pain in his inner thigh muscles and episodes of passing dark red urine. Though the fever subsided after the week, the myalgia lingered and escalated, becoming acutely debilitating. He had no vomiting or diarrhoea, and no dysuria. He had no respiratory symptoms. He had a previous lumbar spinal surgery to correct spinal stenosis. He was a non-smoker. As previously mentioned, he was a fish farmer. He had no history of recent travel.

On examination, he was febrile with a temperature of 38.5 degrees centigrade, heart rate of 83 beats per minute, respiratory rate of 18 breaths per minute, oxygen saturation of 95% on air, and blood pressure of 106/65 mmHg. Chest examination revealed crackles bilaterally in the lung bases while abdominal examination demonstrated marked tenderness in the right upper abdominal quadrant. Examination of his thighs demonstrated marked tenderness in the sartorius muscles bilaterally. There were no abnormal neurological findings in his lower limbs. Additionally, there were no clinical features to suggest meningitis.

Investigations

**Figure 1:**
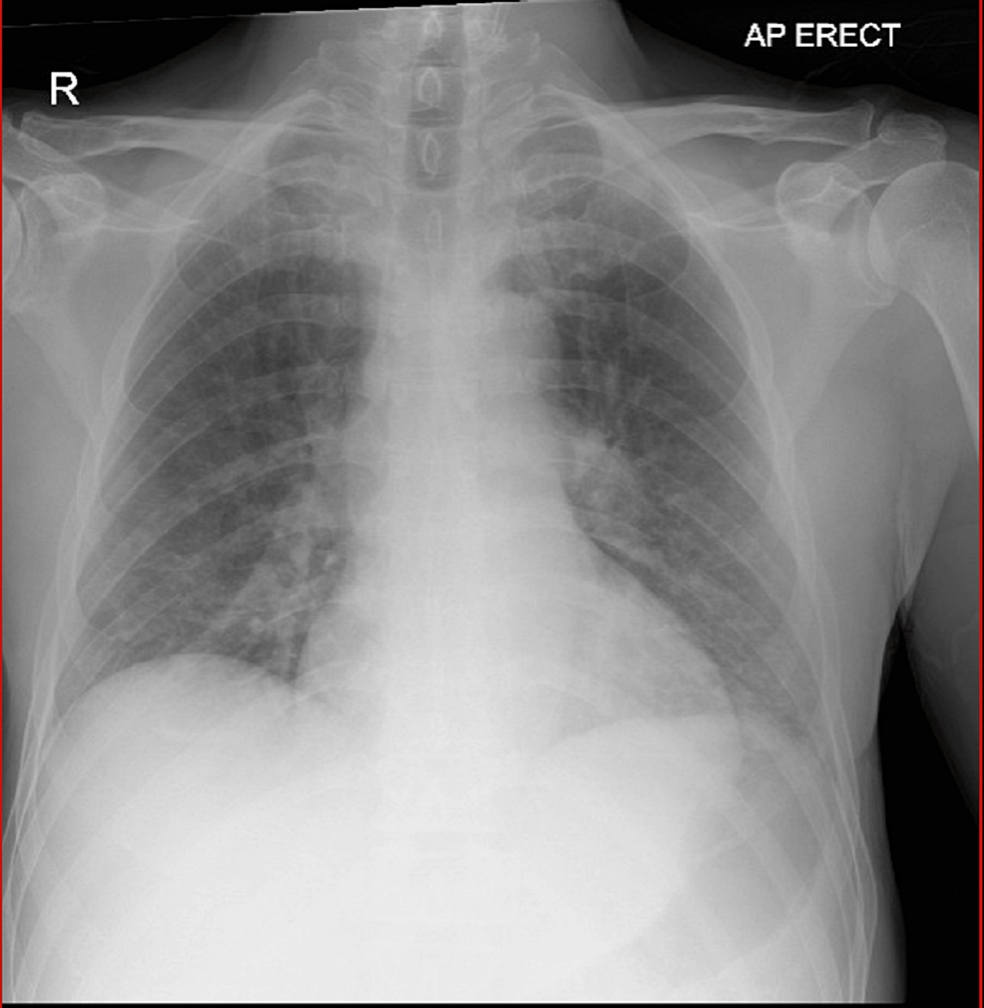
Chest X-ray on admission Generalised vascular prominence noted throughout with the impression of some superadded infection in the left base. Slight left-sided hilar prominence noted.

Laboratory evaluations on admission indicated acute renal impairment, anicteric hepatitis, thrombocytopenia, mild rhabdomyolysis and raised inflammatory markers, all indicating severe sepsis (Table 1). The liver autoimmune antibody screen and hepatitis serology screen were both negative. The antinuclear antibody screen was negative. A chest X-ray on admission hinted at a possible superadded infection in the left lung base (Figure 1). An electrocardiogram demonstrated normal sinus rhythm. An ultrasound scan of the upper abdomen demonstrated normal liver, kidney and pancreas; there were no stones in the gallbladder. Both urine and faecal cultures were negative. A Leptospira PCR report returned positive on the sixth day of admission. Subsequent Leptospira serology (immunoglobulin M (IgM)-ELISA) testing confirmed recent leptospirosis.

**Table 1:** Laboratory blood results during admission Results indicate acute renal impairment, hepatitis, thrombocytopenia, mild rhabdomyolysis and raised inflammatory markers for sepsis.

Parameters	Day 1	Day 3	Day 6	Day 14	Reference Range
White cell count (10^9^/L)	8.4	14.5	16.7	6.9	4.0 - 11.0
Neutrophils (10^9^/L)	7.4	9.4		3.6	1.8 - 7.7
Lymphocytes (10^9^/L)	0.4	3.9		2	1.4 - 4.8
Haemoglobin (g/L)	135	136	135	123	130 - 180
Platelets (10^9^/L)	47	95	197	533	150 - 400
Prothrombin time (ratio)	1.12				0.8-1.25
Activated thromboplastin time (ratio)	0.99				0.8-1.2
C-reactive protein (mg/L)	271	77	29	2	<5
Sodium (mmol/L)	135				133-146
Potassium (mmol/L)	3.8				3.5-5.3
Creatinine (µmol/L)	360	268	152	104	59 - 104
Urea (mmol/L)	21.6	25.8	13.6	5.8	2.5-7.8
Estimated glomerular filtration rate (ml/min)	15	22	44	69	>60
Bicarbonate (mmol/L)	20			26	22-29
Creatine kinase (U/L)	537				40 - 320
Alkaline phosphatase (U/L)	176	212		100	30 - 130
Alanine transferase (U/L)	155	150		37	<41
Total bilirubin (µmol/L)	12				<21
Lactate (mmol/L)	1.9				0.6-2.5
Random glucose (mmol/L)	8.7				3.9-7.0
Blood and urine cultures		Negative			
Leptospira DNA			Positive		
Leptospira serology				Positive	

Treatment

Given the patient's occupational exposure to large bodies of water and potentially rat-infested timber, leptospirosis became a clinical suspicion early on. Management for suspected leptospirosis commenced on day one of admission, involving intravenous ceftriaxone (two grams once daily), intravenous rehydration, and vigilant monitoring of urinary output. By the sixth day of admission, the patient reported wellness and displayed discernible improvement in kidney function and infection markers. The patient was discharged with a seven-day course of oral doxycycline 100 mg daily.

Outcome and follow-up

Follow-up included repeat blood tests a week after discharge, which showed normal inflammatory markers, with his liver enzymes and kidney function back to baseline.

## Discussion

Leptospirosis, as stated, is an exceptionally rare disease, occurring in as low as 0.14 per 100,000 persons in the United Kingdom, thus clinicians may overlook it as a primary differential diagnosis [2]. In this case, our patient presented with myalgia, fever, and episodes of dark red urine. Leptospirosis was suspected because the patient stated that he had heard about the disease through his line of work as a fish farmer and dealt with possible rat-infested timber. This further highlights the importance of thorough history-taking.

The diverse nature of leptospirosis is strikingly evident in its haematological manifestations, further complicating its diagnosis and management. While previous literature has indicated instances of pancytopenia, particularly in cases of leptospirosis with multi-organ involvement, this phenomenon is infrequent [10]. In contrast, our patient exhibited only thrombocytopenia on admission. A notable revelation from a retrospective cohort study highlights the importance of recognising thrombocytopenia in leptospirosis, identifying it as a paramount factor linked to patient mortality. This is attributed to the escalation in bleeding risk, stemming from hindered platelet plug formation and stabilisation, underscoring its pivotal role in disease outcome [11].

Prompt initiation of empirical antibiotic treatment is crucial upon suspecting a diagnosis of leptospirosis. The optimal management strategy includes targeted antibiotic therapy with suitable supportive care to address the multi-organ challenges posed by the infection. It is noteworthy that leptospirosis can have a severe impact, particularly in certain patient demographics. For instance, mortality rates surge to 19.1% in patients presenting with jaundice, 12.1% in those encountering renal failure, and an alarming 60% in patients aged over 60 years [12]. We believe that the prompt initiation of treatment in our patient’s case prevented further deterioration into full-blown icterohemorrhagic leptospirosis.

When it comes to selecting an antibiotic regimen, the severity of the case is a defining factor. In milder instances, choices include doxycycline, amoxicillin, or azithromycin, each with its specific dosages. For moderate to severe cases, a more aggressive approach is warranted, employing antibiotics such as ampicillin, penicillin, ceftriaxone, cefotaxime or azithromycin, particularly for those allergic to penicillin and cephalosporin [13,14]. The importance of liaising with the microbiologist cannot be overemphasized. It is vital to ensure that the specific supportive measures are tailored to the patient, depending on the organ involvement and the overall clinical picture.

## Conclusions

This case report describes a novel case of leptospirosis rarely presenting within the United Kingdom. As leptospirosis shares non-specific symptoms with various other conditions, including influenza, malaria, yellow fever, and dengue fever, establishing clinical suspicion early on, through a detailed clinical history with an emphasis on occupational exposure, is imperative.

A low clinical threshold for suspecting leptospirosis is necessary given a positive clinical history, as in its severe form, clinical deterioration can be rapidly fatal.
